# The Effects of Earthworms on Fungal Diversity and Community Structure in Farmland Soil With Returned Straw

**DOI:** 10.3389/fmicb.2020.594265

**Published:** 2020-12-17

**Authors:** Ke Song, Yafei Sun, Qin Qin, Lijuan Sun, Xianqing Zheng, William Terzaghi, Weiguang Lv, Yong Xue

**Affiliations:** ^1^Eco-Environmental Protection Research Institute, Shanghai Academy of Agricultural Sciences, Shanghai, China; ^2^Department of Biology, Wilkes University, Wilkes-Barre, PA, United States

**Keywords:** earthworms, straw return, biodiversity, fungal community abundance, α-diversity, carbon conversion

## Abstract

**Background:**

To promote the decomposition of returned straw, reduce the incidence of soil-borne diseases caused by returned straw, and accelerate the conversion of straw carbon into soil carbon, we inoculated earthworms into fields with returned straw. The earthworms accelerated straw degradation and promoted carbon conversion. However, the impact of externally inoculated earthworms on the farmland soil ecosystem, especially the structure and the function of its microbial community, remains unclear.

**Methods:**

We analyzed the effects of straw return and earthworms on the diversity of fungal populations and the community structure of dominant fungal taxa in soil by quantifying fungal population size and community composition *via* PCR amplification of internal transcribed spacer genes and 18S rRNA gene sequencing.

**Results:**

The results showed that earthworm inoculation significantly accelerated the degradation of rice straw and promoted the conversion of straw carbon to soil carbon. Both fungal abundance and α-diversity (Sobs and Shannon indices) were higher in the plots with surface straw but without earthworms than in those inoculated with earthworms and in the CK. Principal component analysis indicated that straw return increased the diversity and the abundance of the fungal community, whereas earthworms inhibited this expansion of the fungal community caused by straw return. Interestingly, the overall differences in fungal community composition were smallest in plots with straw return, while the dominant fungal community features in plots inoculated with earthworms were closer to those of the CK.

**Conclusion:**

Generally, straw return stimulated unclassified_K_fungi, *Pseudeurotium*, and *Fusarium* with strong cellulolytic ability. In contrast, the abundances of *Stachybotrys*, unclassified_c_Sordariomycetes, unclassified_f_Lasiosphaeriaceae, and *Schizothecium* were higher in the plots inoculated with earthworms and in the CK. Furthermore, evolutionary analysis showed that the evolution of soil fungal communities tended to diverge after straw return, and the evolutionary directions of fungal species in the plots inoculated with earthworms were similar to those in the CK.

## Introduction

Straw return is an important method for increasing soil organic matter in farmland ecosystems (Benbi and Senapati, 2010). This practice can improve soil structure, increase porosity, reduce bulk density, increase biodiversity, and diversify nutrient supply (Song et al., 2019). As the main sources of cellulase and ligninase, soil microbes play an important role in the degradation of straw (Rui et al., 2009). At the same time, straw return provides abundant carbon for soil microorganisms, thus promoting their growth and activity. It especially increases the proportions of fungi and bacteria (Frey et al., 2003), thus changing the soil microbial community structure (Baumann et al., 2009). Recent research shows that straw return leads to significant changes in the structure and diversity of the soil bacterial ecosystem (Fu et al., 2012; Sun et al., 2013). Soil fungi excrete a comprehensive set of enzymes that allow them to degrade returned straw and thus play an important role in its recycling (Mitchell and Zuccaro, 2006). Bardgett et al. (1993) showed that fungi play a dominant role in the degradation of returned straw, especially in the early stages of plant decomposition when fungi are more active than bacteria and actinomycetes. These findings not only suggest that fungi play an important role in the degradation of returned straw but also show that it is a protracted process (Lu et al., 2015). Therefore, if excessive amounts of straw are returned to the field, its slow rate of decomposition will lead to poor germination, poor seedling growth, and increased incidence of soil-borne diseases (Prasad et al., 2016). These problems have greatly reduced the benefits of straw return.

Earthworms are one of the most common soil animals in terrestrial ecosystems. They can accelerate the decomposition of returned straw by a series of activities, including crushing, feeding, digesting, and burrowing, and thus promote the transformation of fresh straw residues into humus (Six et al., 2004). In particular, the elevated degradative enzymatic activities in earthworm intestines can convert some difficult-to-decompose substances into easy-to-use organic materials, which are then excreted in the wormcasts to facilitate utilization by microorganisms (Hedde et al., 2013). Earthworms can thus significantly increase the rate of decomposition of low-quality straw (high C/N, high lignin and polyphenol content) (Lubbers et al., 2017). In addition, the organic matter and soil are fully mixed during the ingestion–excretion process, which promotes the transfer of surface straw carbon to the deeper layers of the soil and finally enhances soil fertility by increasing the rates of soil nutrient cycling and turnover (Verhulst et al., 2011). Therefore, we have recently inoculated earthworms into fields with returned straw, and these earthworms have promoted the rapid degradation of this straw. The intervention by earthworms obviously accelerated straw degradation. However, how do earthworms affect the soil fungal community in the process of degradation? Do earthworms stimulate fungal growth through their secretions and excrement and increase fungal population diversity and abundance, or do they replace the role of fungi in straw degradation?

Many studies have revealed direct or indirect effects of earthworms on soil microbes (Groffman et al., 2004; Suarez et al., 2004). Some studies suggest that earthworm activities (Curry and Schmidt, 2007) have changed the numbers and the activities of microorganisms (Blouina et al., 2013), which can promote their propagation and growth. The studies by Singer et al. (2001) and Cao et al. (2015) have also shown increases in the numbers of fungal propagules in wormcasts. Other studies have suggested that fungi provided food for earthworms, which reduced the soil fungal populations by eating, dispersing, and killing soil fungal spores and hyphae. Fungal spores can be killed by passage through the earthworm intestinal tract (Moody et al., 1995; Dempsey et al., 2011). Don et al. (2008) proposed that fungi, actinomycetes, and bacteria are all food for earthworms. When these microorganisms pass through the gut, fungal spores are destroyed and actinomycetes are reduced, but the bacteria increase because they can adapt to the anaerobic environment in the gut. Horn et al. (2003) suggested that a small number of fungal bands in the denaturing gradient gel electrophoresis spectrum of wormcasts might be caused by hypoxic conditions in the gut and that intestinal pH (6.9) also had a negative effect on fungi. Schönholzer (1999) suggested that the inhibition of fungi by earthworms is mainly due to the selective uptake and mechanical disintegration of fungal granules in the gut. Recent research on the influence of invasive earthworms on forest soil microbial communities (Price-Christenson et al., 2020) shows that carbon in the leaf litter layer is homogenized between the organic and the mineral horizons by invasive earthworms (*Amynthas* spp.) consuming and eliminating the leaf litter layer. The homogenization and the partial digestion of this carbon shifts the soil microbial community from a fungal-dominated to a bacterial-dominated community. These results demonstrate that earthworm activity has a large impact on soil microorganisms, which may alter their functions and services to the ecosystem.

Although there are many reports on the effects of earthworms on microbial communities (Curry and Schmidt, 2007; Blouina et al., 2013), previous studies have mainly focused on forests and grasslands (Groffman et al., 2004; Suarez et al., 2004; Hale et al., 2006; Dempsey et al., 2011). There are few reports on farmland and, in particular, on the effects of earthworms on fungal population diversity and ecosystem function when straw is returned. Therefore, the mechanisms whereby earthworms change the structure and the function of fungal communities in farmlands are not clear. In view of this, we hypothesized that inoculation of earthworms in a farmland with returned straw would increase the soil fungal population richness and diversity and thus promote the decomposition of returned straw. To test this hypothesis, we assessed the influence of straw return and earthworm inoculation on the abundance, diversity, and evolution of fungal populations and the possible links between earthworm activity and fungal community ecosystem function changes using PCR of internal transcribed spacer (ITS) gene abundances and high-resolution sequencing of 18S rRNA genes based on Illumina MiSeq sequencing.

## Materials and Methods

### Site Description

The experiments were performed at the Samsung Experimental Observatory of the Shanghai Academy of Agricultural Sciences (121°33′47″, 31°41′20″) on Chongming Island, Shanghai, China. The area is located in the northern subtropical zone and has a typical subtropical monsoon climate, with an annual average temperature of 15.3°C. The annual average precipitation is 1,003.7 mm, the annual average sunshine is 2,104 h, and the frost-free period is approximately 229 days. The experimental station mainly grows food crops, and the rice–wheat rotation system has a history of approximately 10 years. The experimental soil is a waterloggogenic paddy soil. Organic matter was 16.35 g⋅kg^–1^, total nitrogen was 0.97 g⋅kg^–1^, alkaline nitrogen was 88.37 mg⋅kg^–1^, available phosphorus was 42.39 mg⋅kg^–1^, and available potassium was 117.66 mg⋅kg^–1^, pH 8.12 (water to soil ratio, 5:1).

### Experimental Materials

Rice (*Oryza sativa* L.) cultivar Hanyou 8 was planted at a seeding rate of 185 kg⋅hm^–2^ and a row spacing of 23 cm. The straw was returned from the rice crop. The C/N of the rice residues was 52.48, with 34.68, 24.55, and 17.84% cellulose, hemicellulose, and lignin content, respectively. All straw from the harvested plants was returned to the field. The part more than 5 cm above the ground was cut into 1–2-cm-sections by an automatic harvester and randomly scattered on the soil surface. The underground debris remained in the soil. The total amount of returned straw was approximately 6 t/hm^2^ (based on the local rice yield of approximately 6 t/hm^2^). The earthworm species used in the experiment was *Metaphire guillelmi*, which was purchased from Shanghai Funian Medicine Co., Ltd. Each earthworm weighed between 2.5 and 3.5 g. Each plot of the treatments with earthworms was inoculated with 50 kg of earthworms, resulting in a density of approximately one earthworm per 3 kg soil in the 0–20-cm soil layer. No crops were planted, no fertilizer was applied throughout the study, and weeding was done weekly to prevent weed growth. In practice, when rice was planted, in order to prevent rice paddy flooding from killing the earthworms, we set up multiple field ridges in the paddies (Additional File 1). Earthworms could migrate to the ridges when the rice paddies were flooded. The measures effectively prevented the earthworms from being drowned (Additional File 1).

### Experimental Design

The experiment was started on November 26, 2017. After the rice was harvested, a total of five treatments were set up according to whether the straw was returned to the field and inoculated with earthworms:

T1: No surface straw with earthworms – Rice straw more than 5 cm above the ground was completely removed from the plot by an automatic harvester. Then, 50 g of earthworms was inoculated evenly, and the plot was not tilled during the experiment.
T2: Surface straw without earthworms – Rice straw more than 5 cm above the ground was cut into 1–2-cm sections by an automatic harvester and randomly scattered on the soil surface. No earthworms were inoculated, and the plot was not tilled during the experiment.
T3: Surface straw with earthworms – Rice straw more than 5 cm above the ground was cut into 1–2-cm sections by an automatic harvester and randomly scattered on the soil surface. Subsequently, 50 g of earthworms was inoculated evenly, and the plot was not tilled during the experiment.
T4: Straw mixed into the soil with earthworms – Rice straw more than 5 cm above the ground was cut into 1–2-cm sections by an automatic harvester, randomly scattered on the soil surface, and mixed with the soil by plowing to a depth of 20 cm in the plot. Subsequently, 50 g of earthworms was inoculated evenly.
T5 (CK): No straw, no earthworms – Rice straw more than 5 cm above the ground was completely removed from the plot by an automatic harvester. No earthworms were inoculated, and the plot was not tilled during the experiment.

Each treatment contained three replicates set up in a randomized block experimental design. Each plot was 40 m × 6 m, and the area was 240 m^2^. The ridges between the plots were covered with a plastic film. The upper edge width was 50 cm, the lower edge width was 70 cm, the underground depth was 60 cm, and the aboveground height was 20 cm to prevent the earthworms from escaping.

### Soil Sampling

Soil samples were collected on January 10, 2018 (56 days after earthworm inoculation) when the straw of the soil surface in T3 (surface straw with earthworms) was almost completely degraded by earthworms. The rice straw on the soil surface was removed before collection, and 0–5-cm-depth surface soil was collected with a 5-cm-diameter corer in each test plot in an “S” pattern. Then, the soil at five points was collected and mixed in each plot to obtain a soil sample. The samples were kept at 4°C prior to animal and plant debris and stones being removed in the lab. The samples were sieved through a 2-mm screen and stored at −80°C before DNA extraction.

### Soil DNA Extraction

Total DNA was extracted from the soil using the FastDNA^®^ SPIN Kit (MP Biomedicals, Santa Ana, CA, United States). Soil DNA was extracted from 0.5 g soil samples according to the kit instructions. The extracted DNA was tested by electrophoresis through 1.0% agarose gels, and the concentration and the quality of the DNA were measured using a NanoDrop ND-2000 analyzer (NanoDrop Technologies, Inc.). Then, the DNA sample was stored at −20°C.

### PCR Amplification

The soil fungal ribosomal gene ITS1–ITS2 spacer was amplified by PCR using the fungal universal primers ITS1F (5′-CTTGGTCATTTAGAGGAAGTAA-3′) and ITS2R (5′-GCTGCGTTCCATCATATGC-3′). After the DNA was validated, it was tested using TaKaRa rTaq DNA Polymerase in 20-μl reactions consisting of 2 μl soil genomic DNA, 0.8 μl each of primer pairs ITS1F and ITS2R (5 μmol/L), 0.2 μl rTaq Polymerase, 0.2 μl bovine serum albumin, 10 ng template DNA, 2 μl 10× buffer, 2 μl 2.5 mM dNTPs, and sterile ddH_2_O to a final volume of 20 μl. An ABI GeneAmp^®^ 9700 PCR machine (Thermo Fisher Scientific, Inc.) was used as follows: initial denaturation at 95°C for 3 min, followed by 35 cycles of 95°C for 30 s, 55°C for 30 s, and 72°C for 45 s, with a final extension at 72°C for 10 min. Three PCR replicates were performed for each sample.

### Illumina MiSeq Sequencing

The recovered product was quantified using a Quantus^TM^ Fluorometer (Promega, United States) after the PCR product was detected by 2% agarose gel electrophoresis. NEXTFLEX^®^ Rapid DNA-Seq Kit was used to build libraries with the following steps: (1) linker ligation, (2) the linker self-ligated fragments were removed by screening with magnetic beads, (3) library template enrichment was performed by PCR amplification, and (4) the final library was obtained by recovering the PCR products with magnetic beads. Sequencing was performed using Illumina’s MiSeq PE300 platform (Shanghai Majorbio Bio-pharm Technology Co., Ltd.). The raw reads were deposited into the NCBI Sequence Read Archive database (accession number: SRP237677).

### Processing Sequencing Data

The raw 18S rRNA gene sequencing reads were demultiplexed, quality-filtered by Trimmomatic, and merged by FLASH with the following criteria: (1) filter reads with quality scores below 20. The sliding window was set to 50 bp, and the back bases were truncated from the window if the average quality score was lower than 20; reads shorter than 50 bp after the quality control and reads containing ambiguous characters were discarded; (2) according to the overlaps between paired-end reads, the paired reads were merged into a sequence, with a minimum overlap of 10 bp; (3) the maximum permitted mismatch ratio of the overlaps was 0.2. Reads that could not be assembled were discarded; and (4) samples were distinguished according to the barcode and primers, and the sequence direction was adjusted, allowing two nucleotide mismatches in primer matching.

Operational taxonomic units (OTUs) with 97% similarity cutoff were clustered using UPARSE (version 7.1)^1^, and chimeric sequences were identified and removed. The taxonomy of each OTU representative sequence was analyzed by RDP Classifier^2^ against the 18S rRNA database – Silva (Release128)^3^, Fungus 18S rRNA database – Unite (Release 7.0)^4^, and Functional gene database – GeneBank (Release7.3)^5^ using a confidence threshold of 0.7.

### Construction of a Phylogenetic Tree

According to the evolutionary relationship between the species in the sample, a phylogenetic tree was selected. High-abundance OTU representative sequences were selected and compared with the NCBI database online. The species were accurately annotated, and a phylogenetic tree was built based on the 16S rRNA gene sequence using the maximum likelihood method. The bootstrap value was set to 1,000 repetitions. Each branch in the phylogenetic tree represents a species. The branches are colored according to the advanced taxonomic level of the species. The length of the branch is the evolutionary distance between the two species, that is, the degree of species difference.

### Statistical Analyses

Statistically significant differences in soil organic carbon (SOC), fungal alpha diversity, and relative abundances of different fungal populations in each sample were tested using one-way analysis of variance (ANOVA) in SPSS 19.0 (SPSS Institute, Inc., 2010). The alpha diversity index, including Sobs (community richness index) and Shannon (community diversity index), was calculated using QIIME software. The dominant fungal taxa were identified according to the method described by Dohrmann et al. (2013). Principal component analysis (PCA) based on Bray–Curtis similarity was performed using the R software package^6^. Cluster analysis of fungal colonies was performed based on Bray–Curtis differences using the “picante” and “vegan” packages in the R environment (B Development Core Team, 2006).

## Results

### Straw Degradation

Earthworm activity significantly accelerated the degradation of rice straw. At the sampling date of the experiment, a visual comparison of T2 and T3, which were both initially covered with surface straw, showed that the straw in T3 was almost completely degraded by earthworms. In contrast, T2 was still mostly covered with straw. At the same time, T4 (straw mixed in soil with earthworms) had no visible undegraded straw. We collected and measured the residual straw in T2 and T3 and found that, after 56 days, the remaining straw in T2 was 73.64%, while it was only 18.57% in T3.

### SOC

**FIGURE 1:**
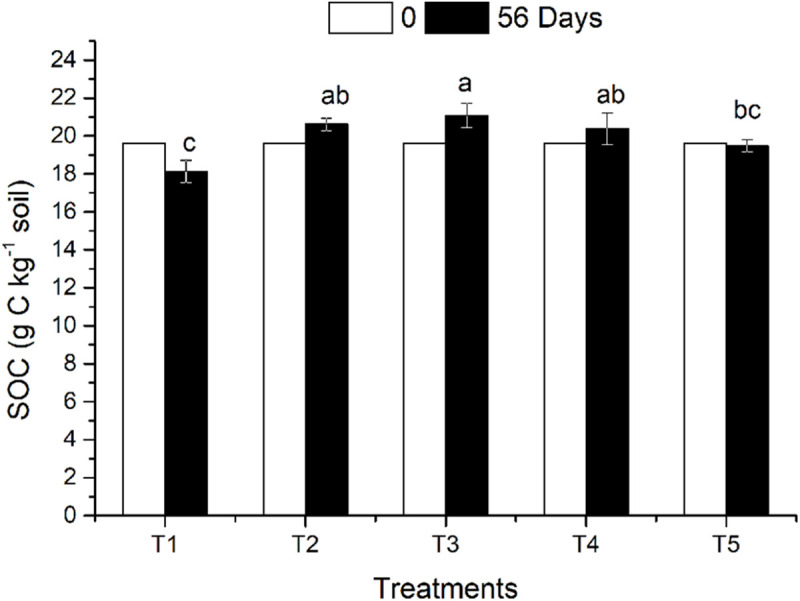
Soil organic carbon in different treatments affected by returned straw and earthworms. The treatments are as follows: T1: no surface straw with earthworms; T2: added surface straw, no earthworms; T3: added surface straw with earthworms; T4: straw mixed into soil with earthworms; T5 (CK): no surface straw, no earthworms. Values are means ± SD, *n* = 3. Treatments indicated by the same letter are not significantly different at *P* ≤ 0.05 on the basis of one-way ANOVA.

The effects of straw return and earthworm activity on SOC in the different treatments are shown in Figure 1. On the 56th day after earthworm inoculation, the SOC decreased slightly in the CK treatment (no returned straw and no earthworms) compared with the initial SOC content. The organic carbon content in the three treatments (T2, T3, and T4) with returned straw was higher than that in the CK treatment. The SOC content of T3 was 8.24% higher than that of T5 (CK), which was a significant difference (*P* ≤ 0.05), while the SOC contents in T2 and T4 were 5.81 and 4.62% higher than that in T5, respectively, which were not significant differences. Earthworm activity had a twofold effect on SOC. Earthworm activity increased SOC content when straw was present. The SOC content in T3 was not only significantly higher than that in T5 but also higher than that in T2 (with straw and without earthworms). When no straw was returned, the SOC content of T1 (with earthworms and without straw) was 6.92% lower than that of T5 (CK), which was a significant difference (*P* ≤ 0.05), indicating that earthworm activity reduced the amount of organic carbon in the soil without returned straw. There was no significant difference in SOC content between T3 (straw on the soil surface) and T4 (straw mixed in the soil), which both had earthworm activity.

### Diversity

**FIGURE 2:**
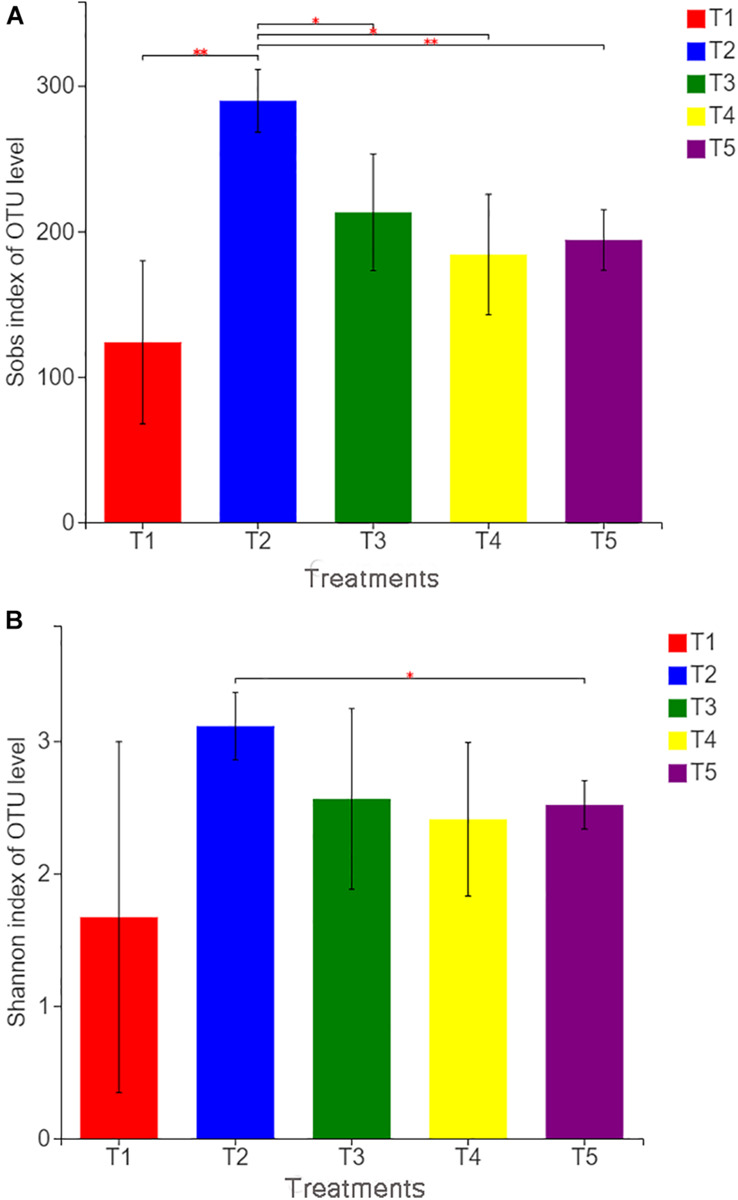
Sobs index **(A)** and Shannon index **(B)** of operational taxonomic unit levels in different treatments. The treatments are described in Figure 1. Values are means, *n* = 3. “^∗^” indicates significant differences at 0.01 ≤ *P* ≤ 0.05, and “^∗∗^” indicates significant differences at 0.001 ≤ *P* ≤ 0.01 on the basis of Student’s *t*-test.

With Illumina MiSeq sequencing of 15 soil samples from five treatments, a total of 553,440 effective sequences were generated, ranging from 37,230 to 59,787 for each sample and 61.71 to 99.10% for the effective sequence. OTU clustering was performed on non-repetitive sequences (excluding single sequences) according to 97% similarity, and 578 OTUs were obtained. The dilution curve of all samples approached a straight line (Additional File 2), and the coverage index reached 99.88%, which indicated that the detection ratio of the sample fungal community was nearly saturated, and the amount of sequencing covered most of the species in the sample. According to the fungal community diversity index (as shown in Figure 2), the Sobs index of T2 was significantly higher than that of other treatments, whereas the Sobs indices of the three treatments (T1, T3, and T4) with earthworms were not significantly different from that of the CK treatment (T5). The Shannon index of T2 was also significantly higher than that of the CK treatment. This finding indicates that the addition of straw in the absence of earthworms increased the observed fungal community richness and the diversity of the soil fungal community. In the treatments (T5 and T1) without straw, the Sob and Shannon indices of T1 with earthworms were lower than those of T5 without earthworms, but there was no significant difference, indicating that the presence of earthworms reduced the fungal community diversity. However, the difference was not significant. In T3 with straw and earthworms, Sob and Shannon indices were not significantly different from those in the CK treatment, indicating that earthworms limited the expansion of soil fungi caused by the straw.

### Principal Component Analysis

**FIGURE 3:**
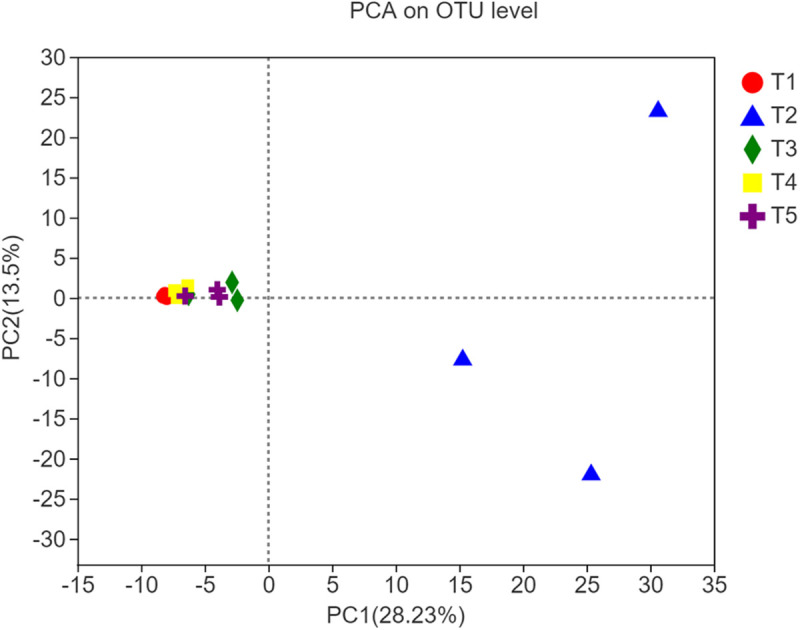
Principal component analysis plot of soil samples from different treatments along principal components 1 and 2, which explained 28.23% and 13.50% of the total variance, respectively.

The different shapes and the color legends in Figure 3 represent the five treatments. According to PCA, at the OTU level, there was a significant difference in fungal community composition between the T2 treatment and the three treatments with earthworms or the CK treatment 8 weeks after straw return and earthworm inoculation. In contrast, there were no obvious differences between the CK treatment and the treatments with earthworms. The measures of dispersion of the fungal community composition of the T2 treatment on the PC1 and PC2 axes were significantly higher than those of the other treatments, and the measures of dispersion in the T1 treatment (with earthworms and without straw) and the T4 treatment (with straw mixed into the soil) were lower than those of the CK treatment. These results indicated that straw return increased the diversity of soil fungal species composition, while earthworms restricted this expansion of the soil fungal species. The interpretation rate of the results for the PC1 axis and the PC2 axis was 28.23 and 13.50%, respectively.

### Hierarchical Clustering Analysis

**FIGURE 4:**
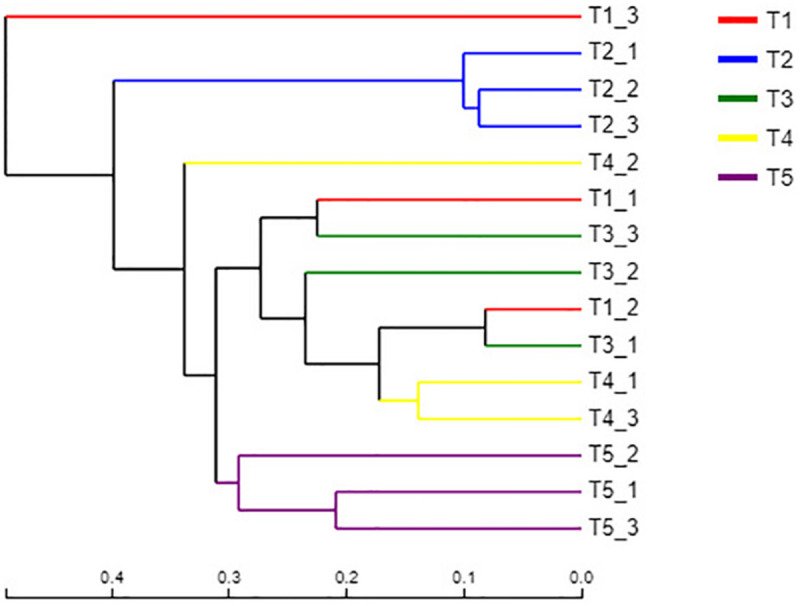
Hierarchical clustering analysis of the fungal communities in the different treatments. The treatments are described in Figure 1. Each branch represents a replication of the treatment. The distances are based on the fungal species and beta diversity distance matrix.

A sample hierarchical clustering analysis was carried out according to fungal species. As shown in Figure 4, all samples tested could be basically divided into three groups. The three replicates of the CK treatment (T5) were classified into one group, and the three replicates of treatment T2 (with returned straw without earthworms) were classified into one group. The remaining three treatments (T1, T3, and T4), which all contained earthworms, were classified into one group. This result indicated that straw return had a significant effect on the fungal community, which was significantly different from the CK treatment. However, the three treatments with earthworms, including T1 without returned straw, T3 with returned straw, and T4 with returned straw mixed into the soil, had no significant differences because of the presence of earthworms, which revealed that earthworm activity offset the effect of straw return on the soil fungal community. However, the fungal community of T1 with earthworms and T5 (CK) without earthworms was significantly different, indicating that earthworm presence caused the community composition of soil fungi to be significantly different from that of the CK treatment.

### Fungal Abundance

**FIGURE 5:**
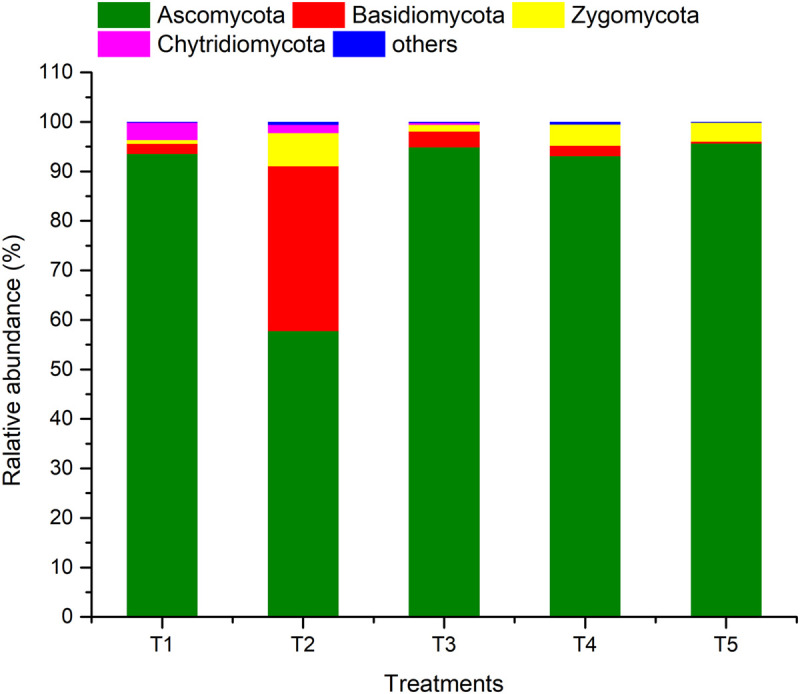
Relative abundances of different fungal phyla in different treatments. The treatments are described in Figure 1.

ARISA-PCR identified a total of one kingdom, seven phyla, 22 classes, 51 orders, 91 families, 145 genera, and 211 species in all of the samples. As shown in Figure 5, members of phylum Ascomycota were the most abundant (57.74–95.68%), followed by Basidiomycota (0.37–33.34%), Zygomycota (0.79–6.67%), and Chytridiomycota (0–3.50%), while the sum of the other fungi was only 0.14–0.57%. The relative abundance of Ascomycota in the treatments (T1, T3, and T4) with earthworms was significantly higher than in the treatment (T2) with straw and without earthworms. The abundance of Basidiomycota in T2 was much higher than in the other treatments, and the abundance of Zygomycota and others was also higher. The abundance of Basidiomycota and Chytridiomycota in the CK treatment (T5) was lower than in the other treatments.

In addition to the effect at the phylum level, a comparison of the CK and T2 treatments (with straw and without earthworms) showed that the presence of straw significantly increased the abundance of fungal taxa such as Eurotiales, Pseudeurotiaceae, Melanosporales, Melanosporaceae, Cystofilobasidiales, Cystofilobasidiaceae, Hypocreales, Ceratostomataceae, and Nectriaceae and significantly decreased the abundance of Sordariomycetes, Hypocreales, Stachybotryaceae, Sordariales, and Lasiosphaeriaceae (Additional File 2). However, in the three treatments (T1, T3, and T4) with earthworms, the only significant increases in abundance compared to the CK were observed in Sordariomycetes, Hypocreales, and Stachybotryaceae, which became the dominant fungal taxa, accounting for 28.47–40.23% of the total. At the same time, earthworms decreased the abundance of fungal taxa such as Sordariales, Chaetomiaceae, Pleosporales, and Sporomiaceae. Compared to the T2 treatment (with straw and without earthworms), the presence of earthworms significantly reduced the abundances of Pseudeurotiaceae, Melanosporaceae, Cystofilobasidiaceae, Ceratostomataceae, and Nectriaceae, whereas it greatly increased the abundances of Hypocreales, Stachybotryaceae, Sordariales, and Chaetomiaceae.

**FIGURE 6:**
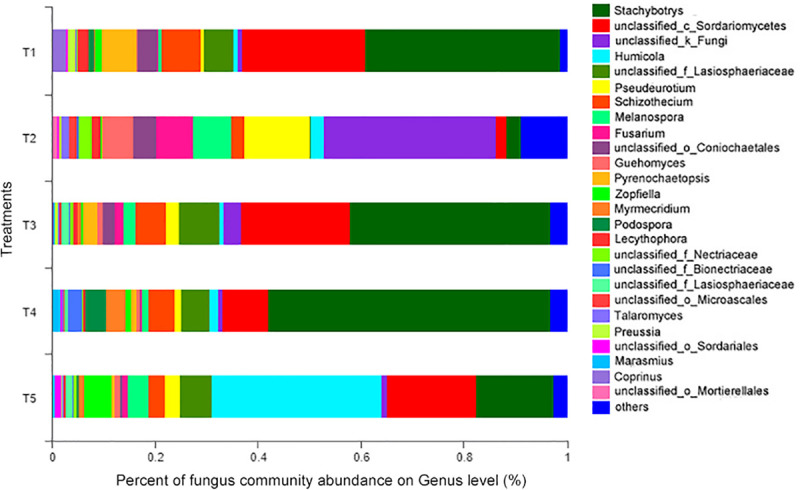
Relative abundances of different fungal genera in different treatments. The treatments are described in Figure 1.

At the genus level (as shown in Figure 6), the 14 most abundant genera accounted for 91.26% of the total sequence data in the CK treatment (T5), 91.01, 92.59, and 88.31% in the T1, T3, and T4 treatments with earthworms, respectively, and only 78.94% in the T2 treatment (with straw and without earthworms). The remaining 131 fungal genera only constituted 7.41–21.06% of the total sequence data. Thus, a few dominant taxa accounted for the majority of the recovered sequences. Among them, as shown in Table 1, the *Humicola* genus had the highest abundance in the CK treatment (no earthworms, no straw) and was much higher than in the other treatments, followed by the genera unclassified_c_Sordariomycetes and *Stachybotrys* and genera with an abundance of more than 5%, including unclassified_f_Lasiosphaeriaceae and *Zopfiella*. In the three treatments with earthworms (T1, T3, and T4), *Stachybotrys* was the most abundant (37.79–54.74%), which was significantly higher than in the CK and T2 treatments, followed by unclassified_c_Sordariomycetes (8.92–23.83%), unclassified_f_Lasiosphaeriaceae (5.36–7.78%), and *Schizothecium* (4.91–7.62%). The abundance of various fungal genera in the T2 treatment (with straw and without earthworms) was very different from that of the CK and the treatments with earthworms. In the T2 treatment, unclassified_k_Fungi was the dominant genus at 33.59%, which was much higher than in the other treatments, followed by the genera *Pseudeurotium* (12.82%), *Melanospora* (7.38%%), *Fusarium* (7.14%), and *Guehomyces* (5.82%). The abundances of these fungal genera were significantly higher than in the CK and the treatments with earthworms. The remaining 8.97% of the total abundance in this treatment came from other fungal genera, which individually accounted for less than 1% of the total. This fraction was much higher than the 2.49% in the CK treatment and in the treatments with earthworms (1.29–3.12%).

**TABLE 1:** The abundances of 14 dominant fungal genera in the different treatments. The treatments are described in Figure 1.

**Dominant genera**	**T1 (%)**	**T2 (%)**	**T3 (%)**	**T4 (%)**	**T5 (%)**
*Stachybotrys*	37.79 ± 33.86a	2.62 ± 0.56b	38.99 ± 22.79a	54.74 ± 31.86a	15.09 ± 7.28a
unclassified_c_Sordariomycetes	23.83 ± 13.78a	2.04 ± 0.60b	21.13 ± 6.40a	8.92 ± 6.59a	17.24 ± 3.13a
unclassified_k_Fungi	0.84 ± 0.33b	33.59 ± 6.94a	3.47 ± 1.94b	0.90 ± 2.66b	1.05 ± 2.33b
*Humicola*	1.02 ± 0.36b	2.48 ± 0.78b	0.82 ± 0.19b	1.75 ± 3.42b	32.99 ± 6.82a
unclassified_f_Lasiosphaeriaceae	6.13 ± 2.56	0.01 ± 1.12	7.78 ± 2.19	5.36 ± 5.90	6.13 ± 12.45
*Pseudeurotium*	0.63 ± 0.20b	12.82 ± 2.28a	2.58 ± 14.8b	1.37 ± 0.58b	3.07 ± 0.67b
*Schizothecium*	7.62 ± 2.61	2.52 ± 1.02	5.84 ± 6.51	4.91 ± 2.62	3.12 ± 1.93
*Melanospora*	0.64 ± 0.88	7.38 ± 3.94	2.33 ± 2.33	1.28 ± 0.17	4.00 ± 9.11
*Fusarium*	0.26 ± 0.08b	7.14 ± 0.70a	1.73 ± 0.60b	0.26 ± 0.43b	1.13 ± 0.54b
unclassified_o_Coniochaetales	3.81 ± 1.78	4.55 ± 1.71	3.17 ± 1.15	2.31 ± 0.36	0.38 ± 0.05
*Guehomyces*	0.13 ± 0.03b	5.82 ± 2.02a	0.98 ± 0.32b	0.67 ± 0.35b	0.01 ± 0.12b
*Pyrenochaetopsis*	6.92 ± 3.08	0.22 ± 0.14	2.76 ± 1.55	1.13 ± 15.67	0.67 ± 1.42
*Zopfiella*	1.23 ± 8.84	0.27 ± 0.08	0.42 ± 0.20	0.87 ± 1.04	5.31 ± 2.57
*Myrmecridium*	0.16 ± 0.29	0.04 ± 0.02	0.58 ± 0.24	3.84 ± 0.71	1.07 ± 0.66

*Values are means ± SD, *n* = 3. Treatments indicated by the same letter are not significantly different at *P* ≤ 0.05 on the basis of one-way ANOVA.*

### Phylogenetic Analysis

**FIGURE 7:**
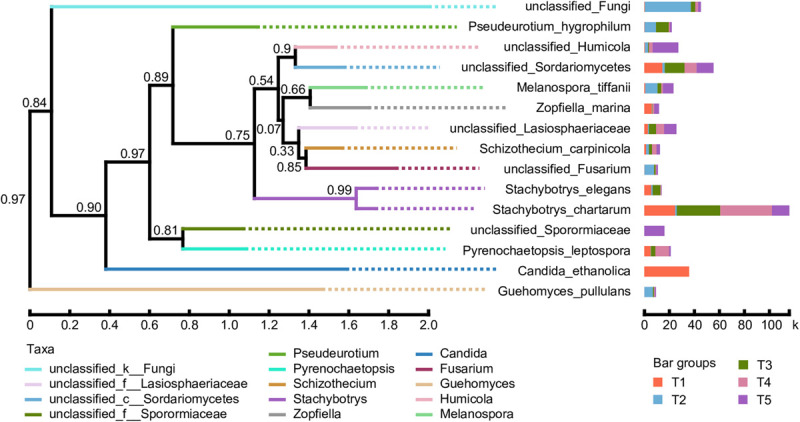
Phylogenetic tree of the dominant fungal species based on 18S rRNA gene sequence comparisons. Bootstrap values from 1,000 replications are indicated at the branches. The abundance of various fungal species in the different treatments is presented after the species name. The treatments are described in Figure 1. The taxa are based on the fungal genus.

A phylogenetic tree of the dominant fungal community was constructed using the approximate maximum-likelihood method by selecting sequences corresponding to the classification information at the species level. As shown in Figure 7, the abundance of various fungal species in the different treatments is presented after the species name. The results showed that the evolution of the soil fungal communities tended to diverge after straw return. Compared with the CK treatment, the proportions of unclassified_Fungi, *Pseudeurotium_hygrophilum*, *Melanospora_tiffanii*, unclassified_*Fusarium*, and *Guehomyces_pullulans* were significantly increased. However, the evolutionary direction of fungal species in the treatments with earthworms was closer to the CK treatment. The key differences are that the proportions of unclassified_*Humicola* and *unclassified_Sporormiaceae* were much higher in the CK treatment, while *Candida_ethanolica* was only detected in the T1 treatments, *Stachybotrys_elegans* had a higher proportion in the T1 and T3 treatments, and *Pyrenochaetopsis_leptospora* was higher in the T1, T3, and T4 treatments.

## Discussion

### Diversity

Fungi are eukaryotic microorganisms that are widely distributed and diverse. Fungi are also the main components of the soil microflora. They play a vital role in maintaining the balance of the ecosystem, supplying plant nutrients, and decomposing organic matter (Hannula et al., 2020). Crop straw is mainly composed of dense carbohydrates coalesced by lignin, cellulose, hemicellulose, etc., which can provide abundant carbon and nitrogen sources for microorganisms during the degradation process. Fungi are the dominant fauna degrading crop straw. Returning straw to the field can increase the diversity and the activity of soil fungi, especially the ratio of fungi to bacteria (Yang et al., 2020b). In this study, the presence of straw significantly increased the Sobs and Shannon indices, indicating that straw return increased the diversity of soil fungi. Returned straw provided an organic carbon source and other nutrients for the fungi. The straw also carried plant-parasitic fungi, and the release of nutrients and the increase in population size increased the community density and diversity of the soil fungi (Holland and Coleman, 1987). However, the Sobs index of the three treatments with earthworms was significantly lower than that of the treatment with straw without earthworms, which shows that rice straw return in the presence of earthworms did not increase the diversity of soil fungi. Thus, the presence of earthworms limited the expansion in numbers and diversity of soil fungi due to straw return. There may be two reasons for this: on the one hand, studies have proven that many fungi are the main food sources of earthworms, especially endogeic and epigeic earthworms. These earthworms feed on fungi and destroy mycelium to inhibit fungal populations and reduce diversity and density (Hoeffner et al., 2018). On the other hand, earthworms change the quality of fungal food resources by feeding on and breaking up organic residues (Gong et al., 2019), which may also affect the succession mode, diversity, and quantity of fungi on plant residues that are being decomposed. For example, studies have shown that, in high-carbon soils, earthworm activity accelerates the consumption of organic matter, reduces the soil microbial biomass, and generally reduces the soil fungus-to-bacteria ratio (Chang et al., 2017). The results of PCA (Figure 3) also showed that the dispersion of fungi on the first principal component axis after adding straw was significantly higher than that of other treatments, while the distribution of fungal populations after inoculation with earthworms was closer to that in the CK treatment, which also showed that the addition of straw increased the fungal diversity, and the inoculation of earthworms limited the expansion of the fungal population to a certain extent.

### Fungal Community Abundance

Soil fungi are all fungi groups that exist in soil, including Zygomycetes, Basidiomycetes, Ascomycetes, and Deuteromycetes. The natural ecological environment, vegetation types, and agricultural management methods will affect the population and community structure of soil fungi (Hu et al., 2019). The straw is decomposed, and plant carbon is fixed in the soil carbon pool by microorganisms, thereby increasing soil organic matter, and soil fungi play a key role in this process. However, how the addition of straw affects the fungal community has not been conclusively determined. Studies have found that the soil fungal community changes significantly after the rice straw is returned to the field, and the number of fungal populations increases significantly (Liu et al., 2019; Yang et al., 2020a). Some strong cellulose-decomposing fungi, such as *Penicillium* (Penicillium) and *Aspergillus* (Aspergillus), have become the dominant taxa. However, studies by Banerjee et al. (2016) and Li et al. (2017) showed that the addition of crop straw increased the number of *Chaetomium*, *Fusarium*, and *Acremonium* spp. with cellulose-degrading ability. The results of this study showed that the addition of straw significantly increased the abundance of unclassified_k_Fungi fungi with cellulose degradation ability and increased *Pseudeurotium*, *Melanospora*, and *Fusarium*. Returning straw to the field changed the community structure of soil fungi and promoted the proliferation of soil fungi related to straw decomposition. However, earthworm activity clearly exerted a negative influence, inhibiting the fungal community and density, resulting in reduced diversity of fungal populations compared with the treatment with straw return only. The dominant fungal community also changed from unclassified_k_Fungi (T2) to Ascomycota *Stachybotrys* (Figure 6). At the same time, the fungi of genera *Pseudeurotium*, *Melanospora*, *Fusarium*, and *Guehomyces* decreased. Most fungi can form a large number of mycelia, and the sporangia or spores are borne on mycelia. Do earthworms reduce fungal diversity and population sizes because their feeding and digestive machinery destroy the hyphae that can form spores and thus limit fungal reproduction and growth? Bonkowski et al. (2000) believed that the mechanisms by which earthworms inhibited fungal communities included their feeding on fungal spores and hyphae. Gange and Brown (2003) suggested that physical interference with fungal hyphae by earthworm burrowing reduced soil organic matter. Lawrence et al. (2003) and Gormsen et al. (2004) also demonstrated that direct consumption and physical interference disrupted fungal colonies, reduced fertility, and had a negative impact on fungal feeding. Seifert et al. (2009) proposed that the soil fungi stopped growing due to the destruction of foraging hyphae. Earthworms also replace the fungi in completing the process of soil C exchange, resulting in the loss of fungal vitality and abundance. Consequently, the diversity of soil fungal communities decreases over time. Although many studies have reported the negative effects of earthworms on soil fungi, some studies have suggested that earthworms have a positive effect on certain soil fungi (Aira et al., 2010; Eisenhauer, 2010; Lavelle, 2012). This is probably because wormcasts are important microsites that provide nutrients and beneficial conditions for fungal growth. The fungi that are not inhibited by earthworm feeding and mechanical destruction may use the nutrients in wormcasts to become the dominant fungal community.

### Fungal Community Functions

Our results show that straw return increased the abundance and the diversity of soil fungi. In contrast, earthworms offset this amplification effect of straw addition. These changes in community abundance and diversity also changed the community structure. Under normal circumstances, the order of fungal development in farmland soil is as follows: fungi that decompose humus → fungi that decompose cellulose → fungi that decompose hemicellulose and pectin (Yang et al., 1992). In our results, the dominant fungal communities in the CK treatment were closely related to the decomposition of humus. In contrast, after straw return, the dominant genera were unclassified_k_Fungi, *Pseudeurotium*, *Melanospora*, and *Fusarium*. Many species within these genera have strong cellulose decomposition activity. Song et al.’s (2018) research proved that unclassified_k_Fungi were the main agents affecting the C/N ratio of decomposed organic matter and were closely involved in cellulose decomposition. However, earthworm activity significantly changed the fungal diversity and influenced the role of fungi in the decomposition of rice straw. The rice straw was almost completely degraded on the 56th day after the addition of straw and earthworms, while most of the soil was still covered with straw without earthworms. This result indicated that earthworms degraded the rice residues much more rapidly than fungi. In this process, the roles of fungi in the decomposition of plant residues and carbon flow were also reduced. Moreover, the results of the hierarchical clustering analysis (Figure 4) showed that the fungal community structure was more similar in the three treatments with earthworms, and there was a significant difference with the treatment without earthworms. This finding shows that the differences in fungal community structure caused by straw return were attenuated by the earthworms. Thus, earthworms played an important role in regulating the structure and the function of the fungal community. Many studies have reported the effects of earthworms on SOC conversion, but these mainly focused on the effects of earthworms on SOC sequestration and mineralization (Zhang et al., 2013; Wachendorf et al., 2014; Lubbers et al., 2017). Our results indicated that the driving force for plant straw degradation when earthworms were present was mainly feeding and digestion by earthworms, and microorganisms played a minor role. The straw was converted into a more easily digestible carbon source, which supplied nutrients to the microorganisms, especially bacteria. This process fueled their growth and reproduction, accelerating the mineralization of unstable carbon sources. In contrast, in the absence of earthworms, straw residues were mainly degraded by microorganisms, and fungi played an important role, but this process took longer (Aira et al., 2019). Wardle (2002) also believed that earthworm activity has led to the transformation of soil ecosystems with fungal-based and slower nutrient turnover into a system dominated by bacteria and rapid turnover of nutrients. This process also indicates that the presence of earthworms alters the role and the function of fungi in SOC conversion.

## Conclusion

In summary, we used PCR and high-throughput sequencing methods to analyze the effects of straw return and earthworm inoculation on fungal community diversity and abundance of the dominant populations. The results demonstrated that straw return in the absence of earthworms stimulated the expansion of soil fungal populations, and the community diversity increased significantly. However, earthworms replaced fungi in straw residue decomposition, which had a negative impact on the dominant fungal community that degraded straw cellulose. These results negated our previous hypothesis that earthworm inoculation in the field of straw return would increase the abundance and the diversity of fungal populations. Instead we demonstrated that earthworms did not accelerate the degradation of straw by stimulating the expansion of soil fungi but, rather, *via* their own feeding and digestion, which improved the efficiency of conversion of straw carbon into soil carbon. In this process, the ecosystem functions and services related to soil biota may also have changed due to changes in the evolutionary trends of fungal populations. Therefore, further research is needed to determine the selective pressure of earthworms on different fungal genera and to assess the functional changes in fungal populations caused by this stress.

## Data Availability Statement

The datasets generated for this study can be found in the NCBI with number SRA: SRP237677, bioproject accession: PRJNA595813.

## Author Contributions

KS, WL, and YX designed this research. KS, YS, and XZ assisted with the design and implementation of the field work. QQ and LS completed the lab experiments and data analysis. KS and YS wrote the manuscript. WT and YX contributed substantially to writing and editing. All the authors have contributed to the final manuscript.

## Conflict of Interest

The authors declare that the research was conducted in the absence of any commercial or financial relationships that could be construed as a potential conflict of interest.

## Footnotes

1 http://drive5.com/uparse/

2 http://rdp.cme.msu.edu/

3 http://www.arb-silva.de

4 http://unite.ut.ee/index.php

5 http://fungene.cme.msu.edu/

6 https://www.r-project.org/
