# Supplementary material for: The Effects of Earthworms on Fungal Diversity and Community Structure in Farmland Soil With Returned Straw
Source: Front Microbiol. 2020 Dec 17;11:594265. doi: 10.3389/fmicb.2020.594265 (PMC7773728; doi:10.3389/fmicb.2020.594265)
Supplement: Supplementary file 1 [file Data_Sheet_1.docx]

**Additional file 1**

The effects of earthworms on fungal diversity and community structures in farmland soils with returned straw

Ke Song^a #^, Yafei Sun^a #^, Qin Qin^a^, Lijuan Sun^a^, Xianqing Zheng^a^, William Terzaghi^b^, Weiguang Lv^a*^, Yong Xue^a*^

^a^Eco-Environmental Protection Research Institute, Shanghai Academy of Agricultural Sciences, Shanghai, 201403, China

^b^Department of Biology, Wilkes University, Wilkes-Barre, PA 18766, USA

***Corresponding authors**

Yong Xue

Tel: +86 18918162296

Fax: +86 021 62202594

E-mail address: [exueyong211@163.com](mailto:exueyong211@163.com)

Weiguang Lv

Tel: +86 18918162056

Fax: +86 021 62202486

E-mail address: weiguanglv1217@163.com

**Supplementary Table S1**. Diversity index Table. The first column is the sample name, and the remaining columns are the values corresponding to the diversity index types in each sample.

| Sample\Estimators | sobs | shannon | coverage | shannoneven |
| --- | --- | --- | --- | --- |
| T4_2 | 244 | 2.977369 | 0.998413 | 0.541619 |
| T3_1 | 176 | 1.817127 | 0.99875 | 0.351442 |
| T5_1 | 177 | 2.685361 | 0.99902 | 0.518795 |
| T3_3 | 254 | 3.168159 | 0.998704 | 0.572145 |
| T1_2 | 165 | 2.090019 | 0.998835 | 0.40933 |
| T5_3 | 232 | 2.539648 | 0.998698 | 0.46627 |
| T5_2 | 212 | 2.323698 | 0.998448 | 0.433802 |
| T2_2 | 269 | 2.851854 | 0.998862 | 0.509741 |
| T1_1 | 158 | 2.732657 | 0.99881 | 0.539774 |
| T3_2 | 232 | 2.709035 | 0.998737 | 0.497368 |
| T4_1 | 163 | 1.816084 | 0.998975 | 0.356532 |
| T1_3 | 71 | 0.193572 | 0.99963 | 0.045411 |
| T4_3 | 177 | 2.444106 | 0.99905 | 0.472186 |
| T2_1 | 332 | 3.364492 | 0.998736 | 0.579572 |
| T2_3 | 295 | 3.126422 | 0.998678 | 0.549751 |


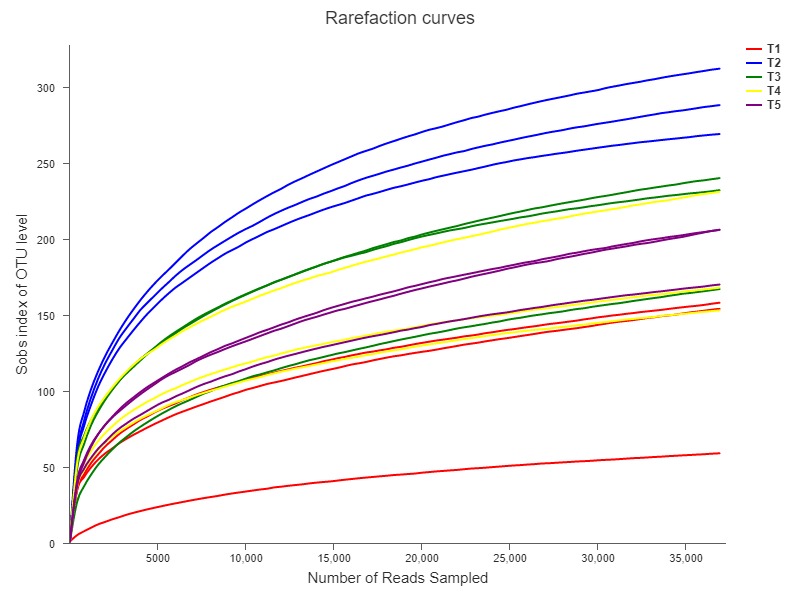


**Supplementary Figure S1. The rarefaction analysis of sequences for all samples.** The abscissa represents the amount of sequencing data randomly sampled; the ordinate, the number of species observed.


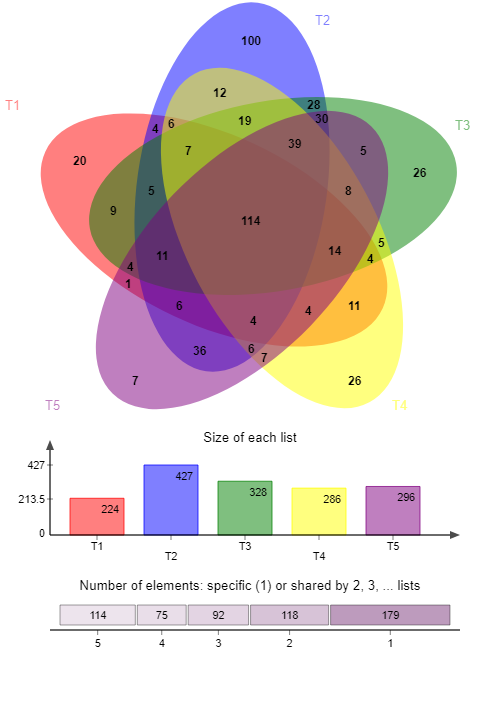


**Supplementary Figure S2. Venn analysis of species.** Different colors represent different treatments, the numbers of overlapping sections represent the number of species common in multiple treatments, and the numbers of non-overlapping sections represent the number of species unique to the corresponding treatments. Treatments are: T1：No surface straw with earthworms，T2：Added surface straw no earthworms，T3：Added surface straw with earthworms，T4：Straw mixed into soil with earthworms，T5（CK）：No surface straw no earthworms.


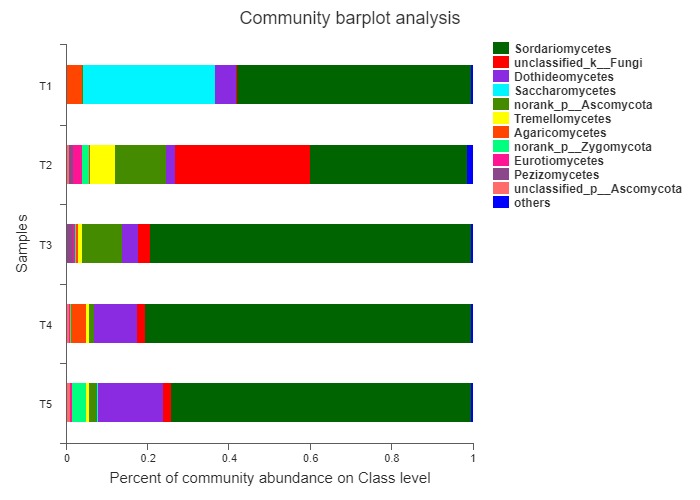


**Supplementary Figure S3. Relative abundances of different fungal Class in different treatments.** The ordinate is the treatments and the abscissa is the proportion of species. Treatments are: T1：No surface straw with earthworms，T2：Added surface straw no earthworms，T3：Added surface straw with earthworms，T4：Straw mixed into soil with earthworms，T5（CK）：No surface straw no earthworms.


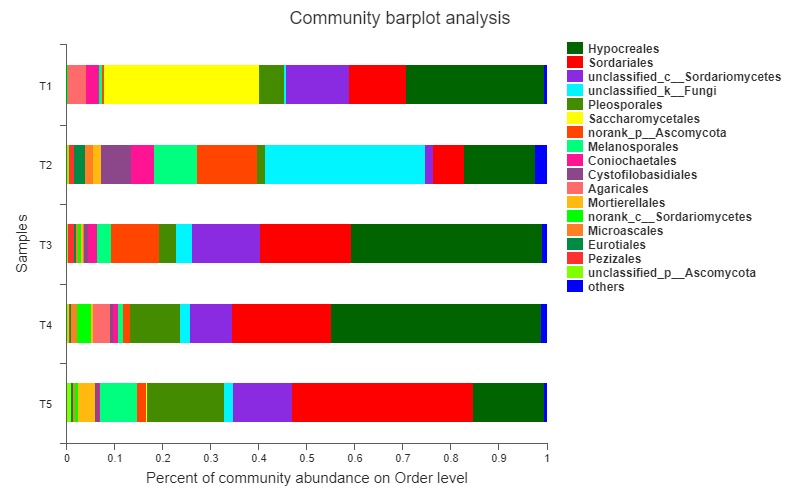


**Supplementary Figure S4. Relative abundances of different fungal Order in different treatments.** The ordinate is the treatments and the abscissa is the proportion of species. Treatments are: T1：No surface straw with earthworms，T2：Added surface straw no earthworms，T3：Added surface straw with earthworms，T4：Straw mixed into soil with earthworms，T5（CK）：No surface straw no earthworms.


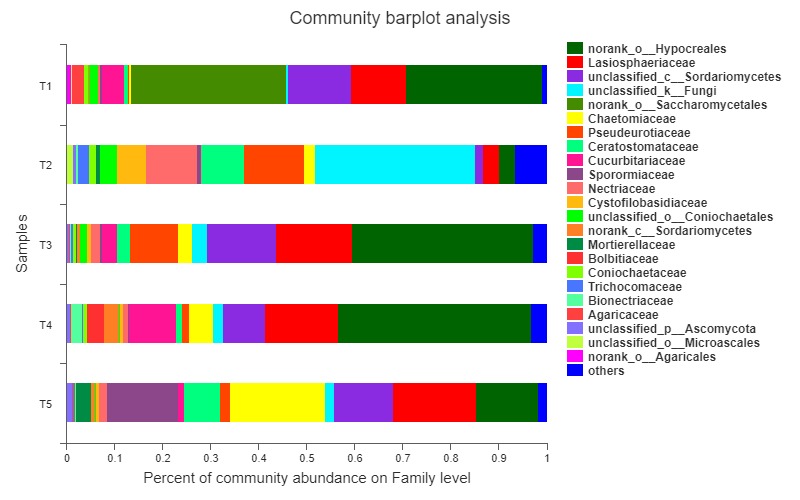


**Supplementary Figure S5. Relative abundances of different fungal Family in different treatments.** The ordinate is the treatments and the abscissa is the proportion of species. Treatments are: T1：No surface straw with earthworms，T2：Added surface straw no earthworms，T3：Added surface straw with earthworms，T4：Straw mixed into soil with earthworms，T5（CK）：No surface straw no earthworms.


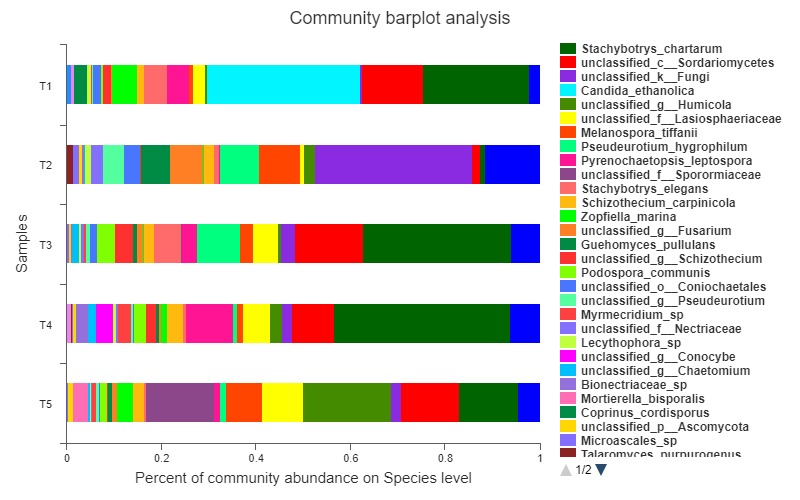


**Supplementary Figure S6. Relative abundances of different fungal Species in different treatments.** The ordinate is the treatments and the abscissa is the proportion of species. Treatments are: T1：No surface straw with earthworms，T2：Added surface straw no earthworms，T3：Added surface straw with earthworms，T4：Straw mixed into soil with earthworms，T5（CK）：No surface straw no earthworms.


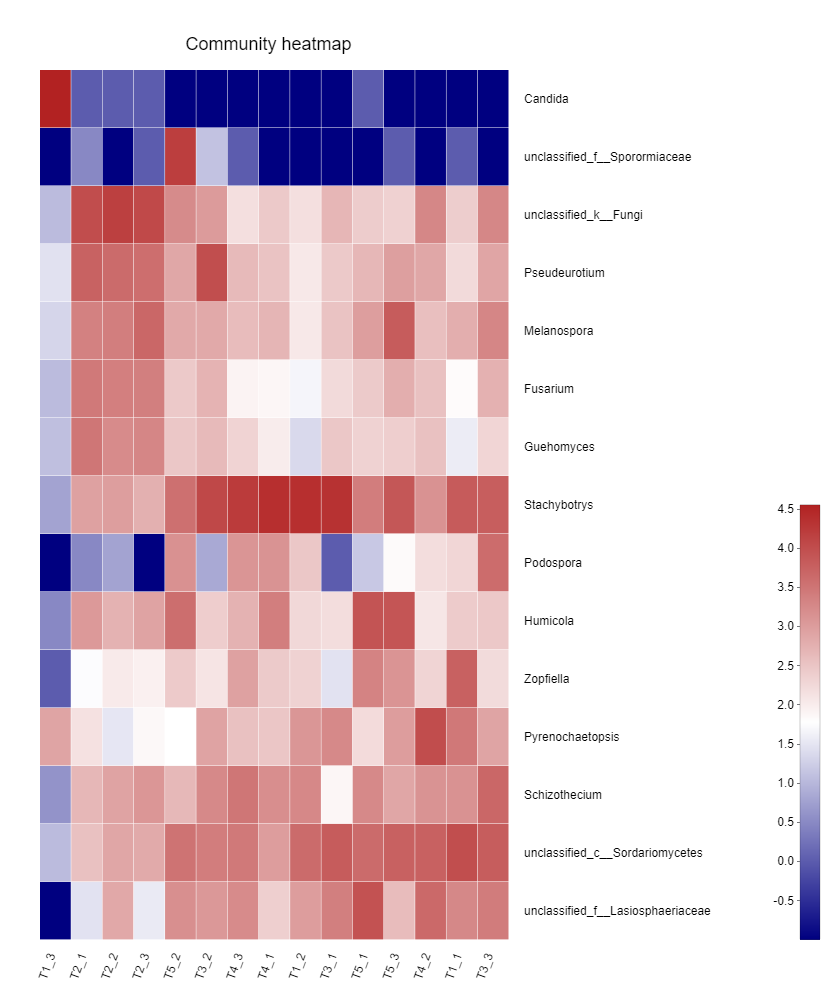


**Supplementary Figure S7. Community Heatmap analysis of fungal genera.** The abscissa is the sample name, and the ordinate is the species name. The color block is used to show the abundance of different species in the sample. Top 15 species of total abundance were shown.


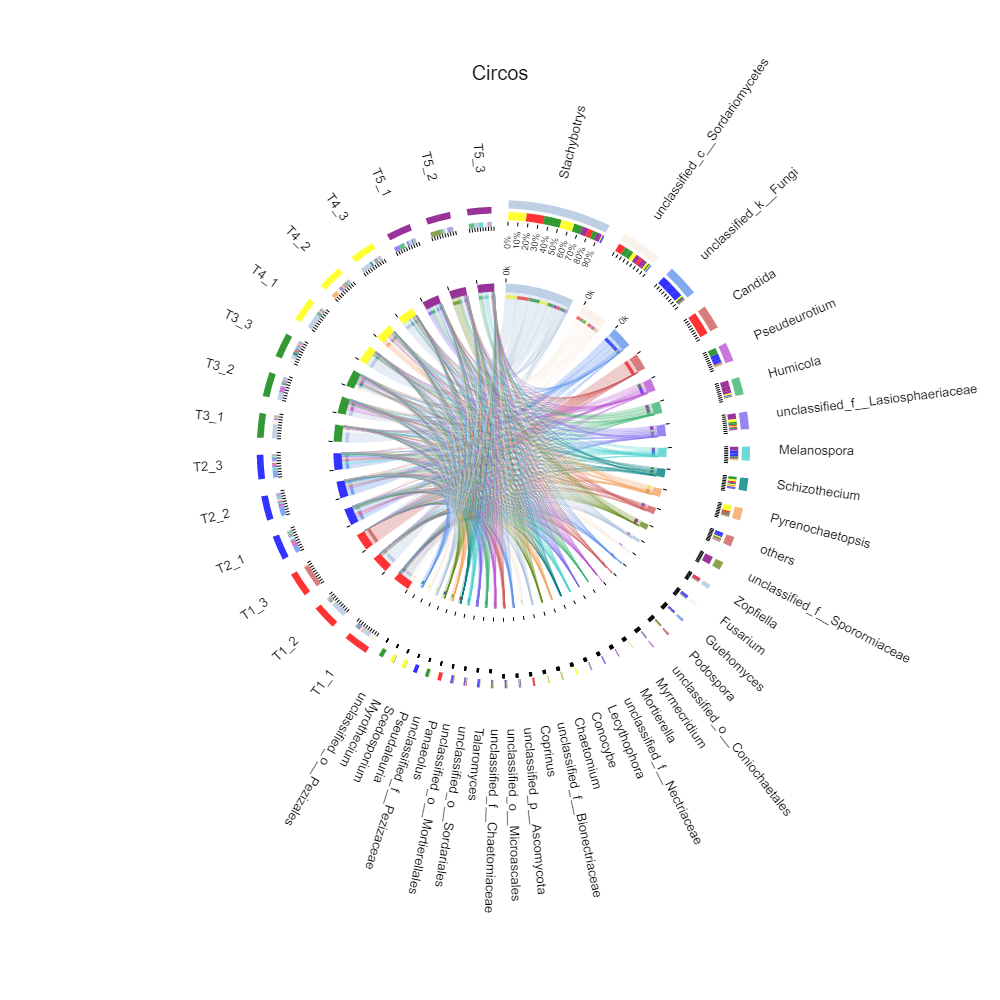


**Supplementary Figure S8. Sample-Species Relations-Circos Analysis.** The small semicircle (left half circle) indicates the composition of the species in the sample. The color of the outer ribbon represents which treatment it came from. The color of the inner ribbon represents the species. The length represents the relative abundance of the species in the corresponding sample. The big semicircle (Right half circle) indicates the distribution ratio of species in different samples at this taxonomic level. The outer ribbon represents the species, the inner ribbon represents the different treatments, and the length represents the distribution ratio of the sample in a species.


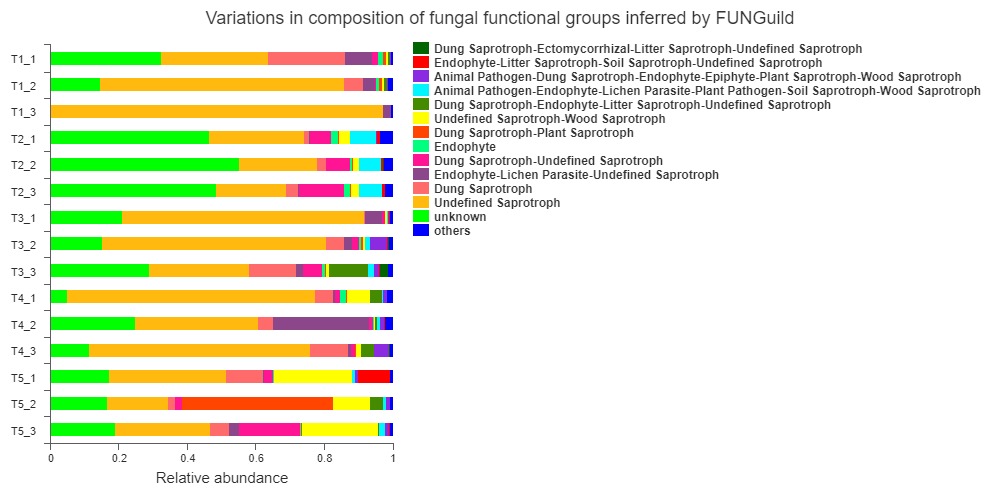


**Supplementary Figure S9. FUNGuild function prediction.** The abscissa is the abundance ratio of Guild in different samples, and the ordinate is different samples.
