# Supplementary material for: The Effects of Earthworms on Fungal Diversity and Community Structure in Farmland Soil With Returned Straw
Source: Front Microbiol. 2020 Dec 17;11:594265. doi: 10.3389/fmicb.2020.594265 (PMC7773728; doi:10.3389/fmicb.2020.594265)
Supplement: Supplementary file 2 [file Data_Sheet_2.docx]

**Additional file 2**

The effects of earthworms on fungal diversity and community structures in farmland soils with returned straw

Ke Song^a #^, Yafei Sun^a #^, Qin Qin^a^, Lijuan Sun^a^, Xianqing Zheng^a^, William Terzaghi^b^, Weiguang Lv^a*^, Yong Xue^a*^

^a^Eco-Environmental Protection Research Institute, Shanghai Academy of Agricultural Sciences, Shanghai, 201403, China

^b^Department of Biology, Wilkes University, Wilkes-Barre, PA 18766, USA

***Corresponding authors**

Yong Xue

Tel: +86 18918162296

Fax: +86 021 62202594

E-mail address: [exueyong211@163.com](mailto:exueyong211@163.com)

Weiguang Lv

Tel: +86 18918162056

Fax: +86 021 62202486

E-mail address: weiguanglv1217@163.com


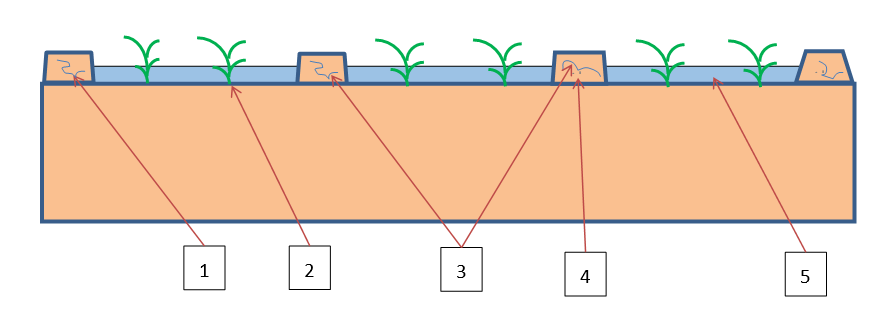


**Supplementary Figure S1. Illustration of rice field inoculation with earthworms.** 1: Tanabe ridge, 2: Rice, 3: Earthworms, 4: Field ridge, 5: Flooding in field.


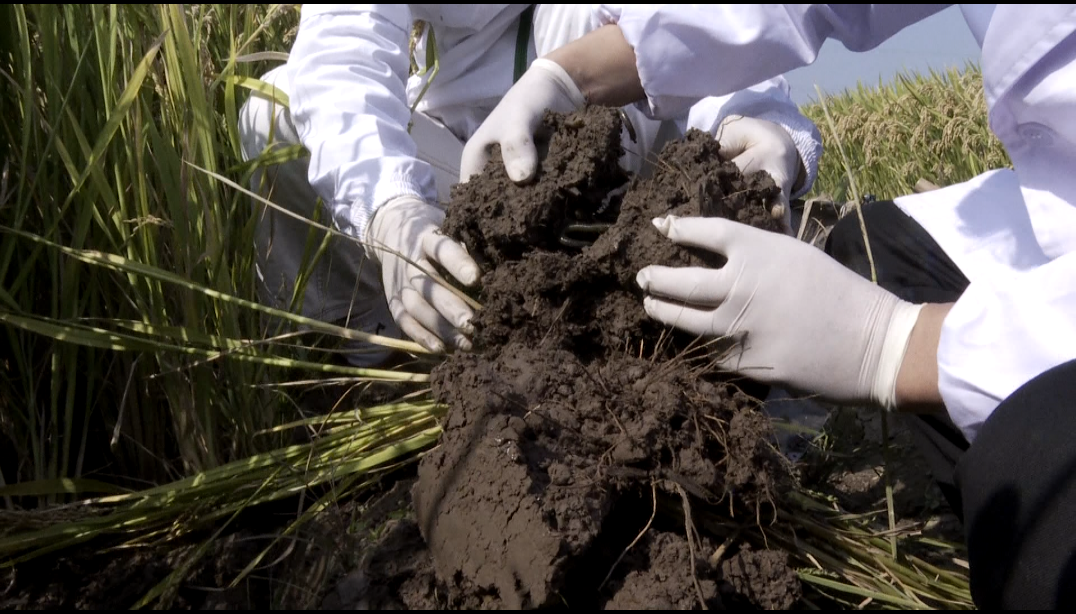


**Supplementary Figure S2 (a). Earthworms in soil of rice root.**


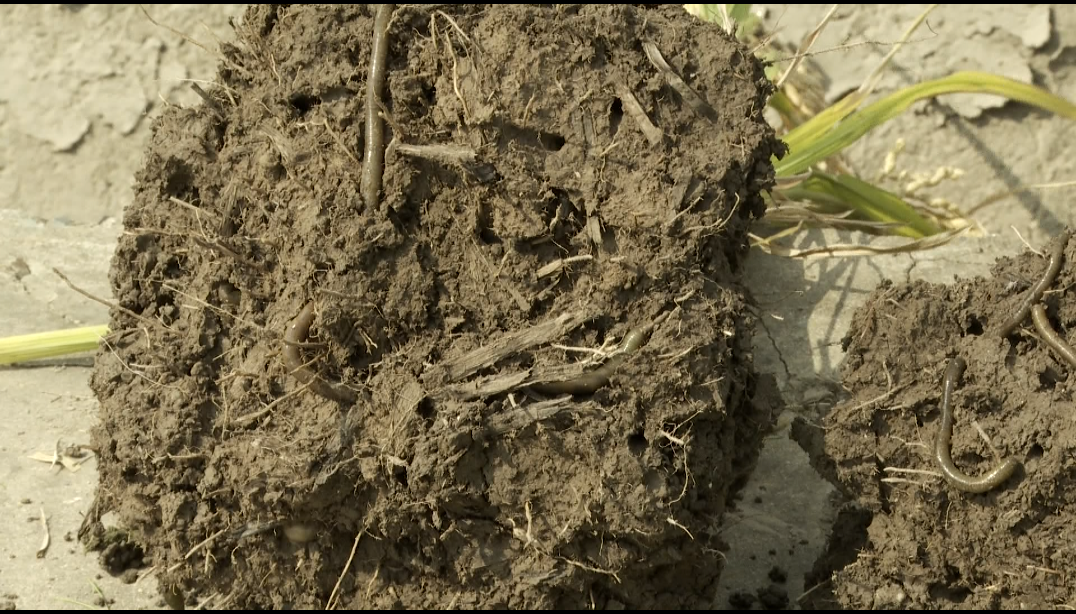


**Supplementary Figure S2 (b). Earthworms in soil of rice root.**


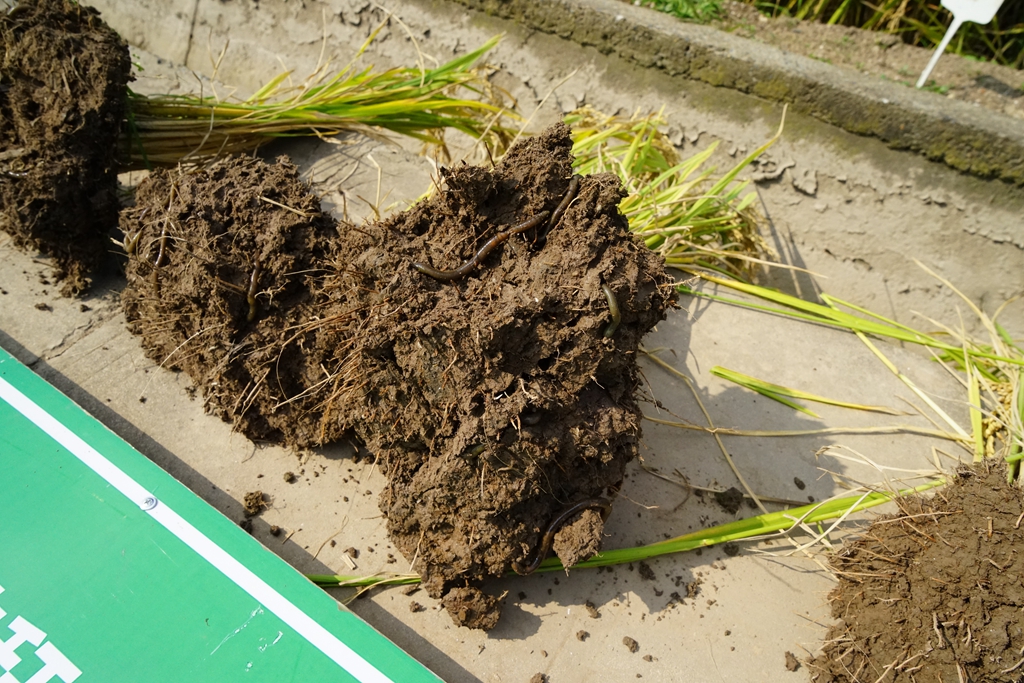


**Supplementary Figure S2 (c). Earthworms in soil of rice root.**


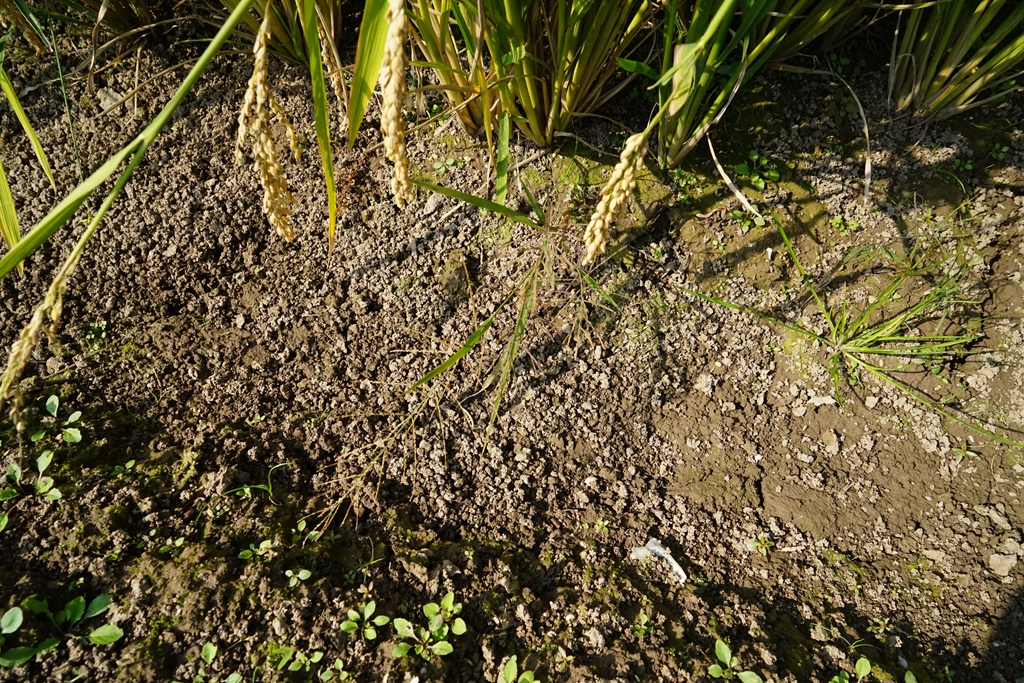


**Supplementary Figure S3. Earthworm cast in rice field.**
